# ASP210: a potent oligonucleotide-based inhibitor effective against TKI-resistant CML cells

**DOI:** 10.1152/ajpcell.00188.2024

**Published:** 2024-06-03

**Authors:** Veronika Nemethova, Petra Babiakova, Boglarka Teglasova, Lucia Uhelska, Andrea Babelova, Michal Selc, Kristina Jakic, Ondrej Mitrovsky, Denisa Myslivcova, Marketa Zackova, Alexandra Poturnayova, Angelika Batorova, Lubos Drgona, Filip Razga

**Affiliations:** ^1^Selecta Biotech SE, Bratislava, Slovakia; ^2^Department of Hematology and Transfusiology, Faculty of Medicine, Comenius University, Bratislava, Slovakia; ^3^Department of Nanobiology, Cancer Research Institute, Biomedical Research Center, Slovak Academy of Sciences, Bratislava, Slovakia; ^4^Centre for Advanced Materials Application, Slovak Academy of Sciences, Bratislava, Slovakia; ^5^Department of Proteomics, Institute of Hematology and Blood Transfusion, Prague, Czech Republic; ^6^Department of Biochemistry, Faculty of Science, Charles University, Prague, Czech Republic; ^7^Department of Condensed Matter Physics, Faculty of Mathematics and Physics, Charles University, Prague, Czech Republic; ^8^Institute of Molecular Physiology and Genetics, Centre of Biosciences, Slovak Academy of Sciences, Bratislava, Slovakia; ^9^Department of Hematology and Transfusiology, Faculty of Medicine, Medical School Comenius University, Slovak Medical University, University Hospital, Bratislava, Slovakia; ^10^Department of Oncohematology, Comenius University and National Cancer Institute, Bratislava, Slovakia

**Keywords:** BCR-ABL1, chronic myelogenous leukemia, oligonucleotide therapeutics, resistance to therapy, tyrosine kinase inhibitors

## Abstract

Clinical experience with tyrosine kinase inhibitors (TKIs) over the past two decades has shown that, despite the apparent therapeutic benefit, nearly 30% of patients with chronic myelogenous leukemia (CML) display primary resistance or intolerance to TKIs, and approximately 25% of those treated are forced to switch TKIs at least once during therapy due to acquired resistance. Safe and effective treatment modalities targeting leukemic clones that escape TKI therapy could hence be game changers in the professional management of these patients. Here, we aimed to investigate the efficacy of a novel therapeutic oligonucleotide of unconventional design, called ASP210, to reduce *BCR-ABL1* mRNA levels in TKI-resistant CML cells, with the assumption of inducing their apoptosis. Imatinib- and dasatinib-resistant sublines of *BCR-ABL1*-positive MOLM-7 and CML-T1 cells were established and exposed to 0.25 and 2.5 µM ASP210 for 10 days. RT-qPCR showed a remarkable reduction of the target mRNA level by >99% after a single application. Cell viability was monitored daily by trypan blue staining. In response to the lack of driver oncoprotein BCR-ABL1, TKI-resistant CML cells underwent apoptosis regardless of the presence of the clinically relevant T315I mutation by *day 5* after redosing with ASP210. The effect was selective for cancer cells, indicating a favorable safety profile for this therapeutic modality. Furthermore, the spontaneous uptake and high intracellular concentrations of ASP210 suggest its potential to be effective at relatively low doses. The present findings suggest that ASP210 is a promising therapeutic avenue for patients with CML who fail to respond to TKI therapy.

**NEW & NOTEWORTHY** Effective treatment modalities targeting leukemic clones that escape tyrosine kinase inhibitor (TKI) therapy could be game changers in the professional management of patients displaying primary resistance, intolerance, or acquired resistance to TKIs. Although delivering authentic innovations today is more complex than ever, we developed a highly potent and safe oligonucleotide-based modality against *BCR-ABL1* mRNA named ASP210 that effectively induces cell death in *BCR-ABL1*-positive TKI-resistant cells while sparing *BCR-ABL1*-negative healthy cells.

## Graphical abstract

**Figure:**
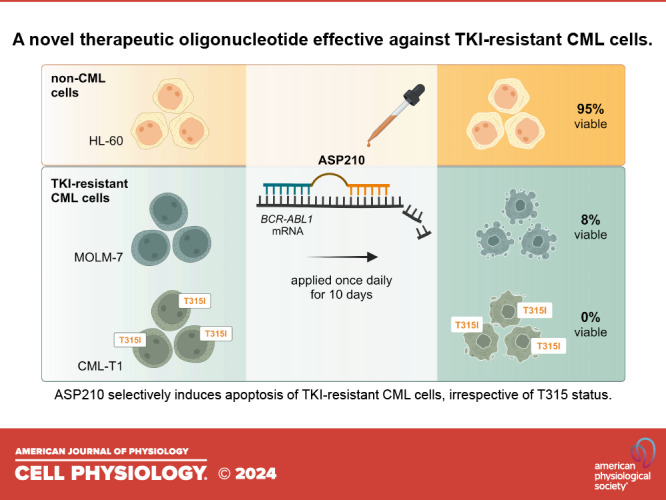

## INTRODUCTION

Chronic myelogenous leukemia (CML) is a clonal hematopoietic stem cell disorder characterized by an increase in myeloid lineage cells at all differentiation stages. CML is associated with a molecular aberration, the fusion gene *BCR-ABL1*, which encodes the chimeric tyrosine kinase oncoprotein BCR-ABL1. Different BCR-ABL1 protein isoforms can be generated according to the position of the *BCR* breakpoint. The e13a2/e14a2 alternative transcripts (or b2a2/b3a2), which result from the juxtaposition of *BCR* exon 13 or 14 with *ABL1* exon 2, produce a 210 kDa protein found in >90% of patients with CML (1).

Advances in our understanding of the molecular basis of CML led to the development of tyrosine kinase inhibitors (TKIs), which have revolutionized the management of CML; the disease currently has a 5-years survival rate of >80% (2). Five TKIs are approved for the treatment of CML: imatinib (IMA), dasatinib (DAS), nilotinib, bosutinib, and ponatinib. The evolution of these drugs for treating CML has been remarkable in a continuous fight against resistance. Nonetheless, despite the massive improvement in CML treatments over recent years, an important minority of patients (20%–30%) display intrinsic or acquired resistance to treatment during the disease course (3). Primary resistance implies failure to achieve time-dependent endpoints of complete hematological, cytogenetic, and major molecular response upon initiation of TKI therapy, whereas secondary (acquired) resistance is defined as the loss of response.

Resistance to targeted therapies is a complex and multifactorial process that culminates in the selection of a cancer clone with the ability to evade treatment. The TKI resistance mechanisms are usually subdivided into BCR-ABL1-dependent and independent mechanisms. BCR-ABL1-dependent resistance is reliant upon mechanisms that subvert effective BCR-ABL1 kinase inhibition. Among these, BCR-ABL1 mutations impair drug binding by either decreasing the affinity of TKIs for the binding domain or by changing the conformation of the oncoprotein, and overexpression of *BCR-ABL1* leads to resistance by increasing the concentration of oncoprotein that needs to be inhibited by the TKI (3, 4).

The occurrence of point mutations on the *ABL1* kinase domain is the most common TKI resistance mechanism. The most frequently detected mutation (4%–20%) among resistant patients is T315I (isoleucine replaces threonine at position 315 of BCR-ABL1), which is called a “gatekeeper” mutation (3, 4). T315I confers resistance to all TKIs approved for frontline therapy and is only sensitive to ponatinib (5). However, T315I-inclusive compound mutations, defined as a *BCR-ABL1* allele with two or more mutations including T315I, have been associated with ponatinib failure in advanced phase CML.

The current strategies for addressing TKI resistance are focused on improving the potency and specificity of the drugs that target BCR-ABL1, as well as overcoming resistance driven by mutations in the *BCR-ABL1* oncogene. Alternative approaches to address current treatment challenges may be necessary for patients who fail to respond to TKIs, and novel therapeutic modalities capable of targeting leukemic clones that escape TKI therapy could be game changers in the professional management of these patients.

Unlike protein inhibitors, oligonucleotides have a distinguishing feature in that they can halt the process of producing a pathological protein before it even begins. Oligonucleotide therapeutics have generated high expectations because of their potency and have enjoyed remarkable success in the clinic in recent years (6). Because diseases directly associated with a genetic aberration are amenable to oligonucleotide-based intervention, antisense systems for *BCR-ABL1* suppression represent a promising treatment option for CML. The use of junction-specific antisense oligonucleotides for the treatment of CML has been tested by several research groups, leading to a reported 25%–80% reduction of *BCR-ABL1* transcript levels in preclinical studies (7–13). Irrespective of the successful silencing of *BCR-ABL1* in most studies, drug promiscuity in terms of their interaction with homologous off-targets places severe limitations on this approach, and off-target-related toxicity contributes to their poor toxicological profile (14). An unconventional approach first reported in 2017 considered the steric and thermodynamic aspects of the interactions between oligonucleotides and target RNA as additional parameters in the design of therapeutic oligonucleotides, which significantly improved their specificity and, hence, safety (15).

**Figure 1.**
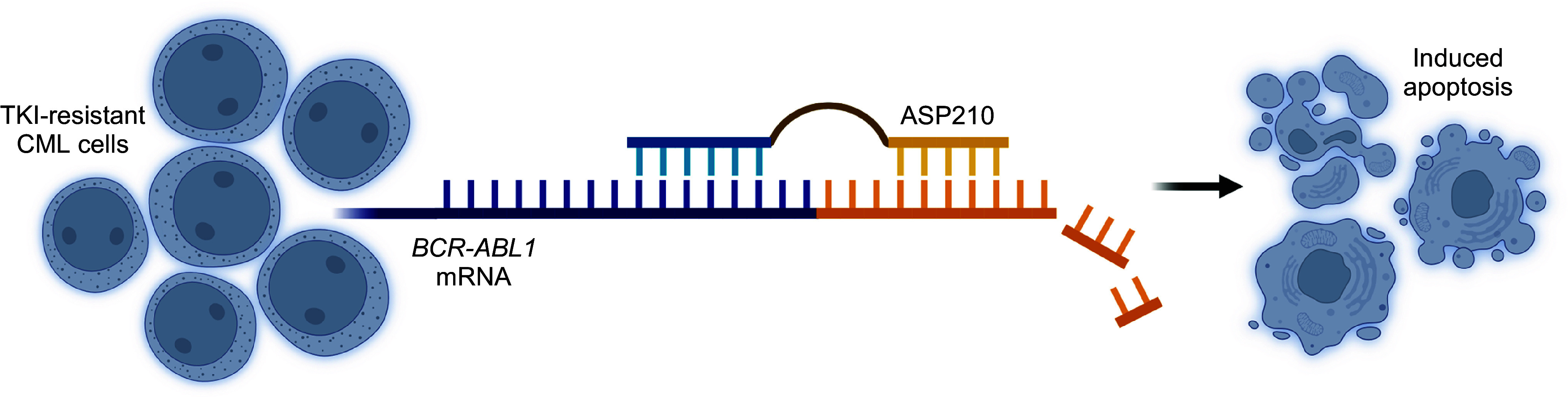
Schematic representation of the annealing of ASP210 to the target *BCR-ABL1* mRNA in the proximity of the fusion junction (blue and orange). The DNA-RNA duplex is recognized by RNase H which catalyzes its enzymatic cleavage. This prevents further synthesis of the BCR-ABL1 oncoprotein. In response to the lack of driver oncoprotein, CML cells undergo apoptosis. CML, chronic myelogenous leukemia. Figure created with BioRender.com.

This proprietary platform, now called ESiNAR-X, served as the basis for the development of a highly specific oligonucleotide-based lead pharmacological modality against *BCR-ABL1*, namely, ASP210. The idea was to create a structure that can attach and block two sites of the target mRNA simultaneously (16). Based on the fact that these mRNA sequences are not exactly next to each other, but maintain a specific distance between them, a modality was proposed that consists of two mRNA site-specific oligonucleotides connected by a linker of suitable length. This structure reduces the likelihood that the two connected oligonucleotides will bind to and act against off-target mRNA molecules, thereby preventing disruption of their function and thus toxic side effects. ASP210 was designed to simultaneously bind to the adjacent sequences of the fusion junction site, providing exquisite selectivity toward the target *BCR-ABL1* mRNA (Fig. 1).

The present study demonstrates the effective reduction of BCR-ABL1 levels in IMA and DAS-resistant (further referred to as IMA-R or DAS-R) MOLM-7 and CML-T1 cell lines using ASP210, irrespective of their T315I status. The cytoreductive potential of ASP210 in terms of its ability to induce apoptosis was confirmed in all TKI-resistant CML cells tested.

## MATERIALS AND METHODS

### Inhibitor ASP210

ASP210, a 34mer phosphorothioate DNA with an internal poly(ethylene glycol) (PEG) linker, was designed by the authors Razga and Nemethova and synthesized by Selecta Biotech (Bratislava, Slovakia) using an automated H28 DNA synthesizer (K&A Laborgeraete, Schaafheim, Germany). The oligonucleotide was labeled with Cyanine5 and biotin at the 5′ and 3′ ends, respectively, to generate 5′-Cyanine5- GCTACTGGCCGCTGAAG(PEG12) CCTTATTGATGGTCAGC-biotin. The high-performance liquid chromatography-purified compound was stored at −20°C in either lyophilized form or dissolved in nuclease-free water.

### Cell Cultures

Human *BCR-ABL1*-positive MOLM-7 (kindly provided by Dr. J. Minowada, Fujisaki Cell Center, Japan) and CML-T1 (DSMZ, Braunschweig, Germany) cell lines expressing the target e13a2 fusion transcript, and *BCR-ABL1*-negative HL-60 leukemia cells (ATCC, Manassas, VA) were used in this study. Cells were maintained in RPMI medium (Gibco, Waltham, MA), supplemented with 10% fetal bovine serum (FBS; Gibco) and 1% penicillin/streptomycin (P/S; Gibco) at 37°C with 5% CO_2_.

### Establishment of TKI-Resistant Sublines

TKI-resistant sublines were prepared from parental MOLM-7 and CML-T1 cells following the protocol by Mahon et al. (17). Each experiment was performed three times. Briefly, cells resistant to either IMA (Cayman Chemical, Ann Arbor, MI) or DAS (Merck, Darmstadt, Germany) were developed by prolonged exposures to increasing concentrations of TKI (1 nM–100 µM IMA; 1 pM–100 nM DAS). All cells were incubated in RPMI medium, supplemented with 10% FBS and 1% P/S at 37°C with 5% CO_2_. TKI-resistant cells were maintained in the presence of either 2 µM IMA or 2 nM DAS.

### Mutational Analysis of *BCR-ABL1* Kinase Domain

#### Reverse transcription.

Total RNA was extracted using the NucleoSpin RNA Plus kit (Macherey-Nagel, Dueren, Germany) according to the appropriate protocol. cDNA was synthesized using oligo(dT) primer and SuperScript II transcriptase (Invitrogen, Waltham, MA) following the manufacturer’s instructions on a Mixing Block MB-120 (Bioer, Hangzhou, PR China).

#### Nested RT-PCR.

Long-range nested RT-PCR was performed to amplify *BCR-ABL1* cDNA using the following primers: Step 1: BCR-F 5′- TGACCAACTCGTGTGTGAAACTC-3′ and ABL1-R 5′- TCCACTTCGTCTGAGATACTGGATT-3′; Step 2: ABL1-F 5′- CGCAACAAGCCCACTGTCT-3′ and ABL1-R 5′- TCCACTTCGTCTGAGATACTGGATT-3′ (IDT, Coralville, IA). Step 1 involved initial denaturation at 95°C for 2 min and 40 cycles of denaturation (95°C for 1 min), annealing (60°C for 1 min), elongation (72°C for 3 min), and a final 10-min extension step at 72°C. Step 2 included initial denaturation at 95°C for 2 min and 50 cycles of denaturation (95°C for 1 min), annealing (60°C for 1 min), elongation (72°C for 3 min), and a final 10-min extension step at 72°C. PCR was performed on a CFX96 Touch Real-Time PCR Detection System (Bio-Rad, Hercules, CA) using gb PCR master mix (Generi Biotech, Hradec Kralove, Czech Republic).

#### Sequencing.

Amplicons were separated on a 2% agarose gel containing MIDORI Green Advance nucleic acid stain (Nippon Genetics Europe, Düren, Germany). Appropriate bands were cut out and purified with the QIAquick Gel Extraction kit (Qiagen, Hilden, Germany). Sanger sequencing was performed on ABI PRISM 3500 Genetic Analyzer using Big Dye Terminator 3.1 kit (Applied Biosystems, Waltham, MA). The resultant sequences were analyzed and aligned using Chromas 2.31 and Basic Local Alignment Tool.

### Cytogenetics

Cells were processed according to standard cytogenetic procedures for fluorescence in situ hybridization (FISH) and multicolor FISH (mFISH) and analyzed using the Vysis LSI BCR/ABL Dual Color, Dual Fusion Translocation Probe (Abbott, Chicago, IL) and 24 XCyte mFISH kit (MetaSystems, Altlussheim, Germany). Mitoses and 200 nuclei were analyzed using an AxioImager Z1 fluorescence microscope (Carl Zeiss, Oberkochen, Germany) and the Isis computer analysis system (MetaSystems). Findings were described according to ISCN 2016.

### Treatment of Cells

Before experiments, the amount of FBS was gradually decreased to 2% for HL-60 and IMA-resistant MOLM-7 and CML-T1 cells, and 5% for DAS-resistant MOLM-7 and CML-T1 cells to slow cell growth and avoid hyperproliferation.

For confocal microscopy and quantitation of cellular uptake, HL-60 and TKI-resistant MOLM-7 and CML-T1 cells, seeded in 48-well plates in 200 µL medium at a density of 2 × 10^5^ cells/well, were exposed to 0.25, 2.5, or 5.0 µM ASP210 and incubated at 37°C and 5% CO_2_ for 24 h. After treatment, cells were pelleted in a microtube at 200 *g* for 5 min and dispersed in 110 µL phosphate-buffered saline (PBS; Oxoid, Lenexa, KS).

To analyze the efficacy of ASP210 in terms of its ability to suppress *BCR-ABL1* and induce cell death, HL-60 and TKI-resistant MOLM-7 and CML-T1 cells, seeded in 48-well plates in 200 µL medium at a density of 2 × 10^4^ cells/well, were treated with 0.25 or 2.5 µM ASP210 once daily and incubated at 37°C and 5% CO_2_ for 1–10 days. After treatment, cells were pelleted in a microtube at 200 *g* for 5 min and dispersed in 110 µL PBS. Five independent experiments were performed, each in duplicate. Data are reported as the mean values ± SE.

### Confocal Microscopy

After treatment, 50 µL of cell suspension in PBS was used for confocal microscopy. Cell membranes were stained with wheat germ agglutinin, Texas Red-X Conjugate membrane stain (Invitrogen) and visualized at 615 nm. Intracellular ASP210 was visualized at 680 nm using a Leica TCS SP8 AOBS confocal microscope (Leica Microsystems, Wetzlar, Germany).

### Quantitation of Cellular Uptake

After treatment, cells were washed and 50 µL of cell suspension in PBS was centrifuged at 200 *g* for 5 min. Cells were counted and lysed using 40 µL lysis buffer containing RIPA (Serva, Heidelberg, Germany) with 1% SDS (Merck) at 4°C for 30 min, and then briefly sonicated using a Sonopuls Mini 20 instrument (Bandelin, Germany). A 10 µL sample of cell lysate was loaded onto a 6% polyacrylamide gel and subjected to electrophoresis (110 V, 40 min, room temperature). ASP210 was visualized at 680 nm and its amount was determined based on the fluorescence signal of a reference sample containing a defined number of ASP210 molecules and results were expressed per cell.

### Analysis of *BCR-ABL1* Suppression

#### Isolation of RNA.

Total RNA was isolated from cells using the phenol-chloroform method (TRIzol, Invitrogen) as recommended by the manufacturer and then purified and treated with DNase I (Thermo Fisher Scientific, Waltham, MA). RNA concentration and purity were measured on a BioDrop µLITE+ spectrophotometer (Harvard Bioscience, Holliston, MA).

#### Real-Time RT-PCR.

Real-time RT-PCR assays for quantitation of fusion *BCR-ABL1* (Hs03024541_ft) and housekeeping *GAPDH* (Hs04420632_g1) transcripts were purchased from Thermo Fisher Scientific. Real-time RT-PCR was carried out in a final volume of 10 µL containing 10 ng total RNA using AmpliTune 1-step RT-qPCR Probe Mix (Selecta Biotech) according to the instructions for users. Amplification was carried out on an AriaMX automated PCR instrument (Agilent, Santa Clara, CA). Briefly, RNA was reverse transcribed at 50°C for 10 min, whereas subsequent cDNA amplification involved the initial activation of DNA polymerase at 95°C for 3 min and 40 cycles of denaturation (95°C for 15 s) and annealing/elongation (58°C for 30 s). All data were normalized against *GAPDH*. Data from treated cells were also normalized against controls and analyzed by the 2^−ΔΔCt^ method.

### Analysis of Cell Viability

After treatment, 10 µL cell suspension in PBS was used for viability assessment by Trypan blue staining (Merck) following the manufacturer’s instructions using a CellDrop FL automated cell counter (DeNovix, Wilmington, DE). Cell viability was normalized to controls, i.e., to untreated cells on the respective day. Cell counts were also used for normalization of internalized ASP210 per cell.

### Western Blot

Within a representative experiment, IMA-R CML-T1 cells, seeded in 96-well plates in 200 µL medium at a density of 2 × 10^4^ cells/well, were treated with 2.5 µM ASP210 once daily and incubated at 37°C and 5% CO_2_ for 1–8 days. After treatment, cells were pelleted in a microtube at 200 *g* for 5 min, washed with PBS, and lysed using 30 µL cell lysis buffer with PMSF (Cell Signaling Technology, Danvers, MA) following the manufacturer’s instructions. Protein concentrations were measured by Bradford protein assay (Bio-Rad), and equal amounts of proteins (50 µg) were incubated at 95°C for 5 min with SDS sample buffer (Merck) before being loaded onto a 7.5% SDS-polyacrylamide gel. After electrophoresis (110 V, 2 h, 4°C), proteins were transferred (350 mA, 1 h, 4°C) onto a 0.2 µm nitrocellulose Amersham Western blotting membrane (Merck). After blocking the membrane in 5% nonfat-dried milk (Merck), it was incubated with either anti-BCR-ABL1 e13a2 junction-specific (3908S; Cell Signaling Technology) or anti-β-actin (A1978; Merck) antibody, both diluted 1:1,000 in 5% nonfat-dried milk. After 24 h, the membrane was incubated for 1 h with a goat anti-mouse IgG polyclonal secondary antibody (Thermo Fisher Scientific) diluted 1:10,000 in nonfat-dried milk. Proteins were visualized using the Odyssey Imaging System (LI-COR Biosciences, Lincoln, NE) and evaluated using the Image Studio Software (LI-COR Biosciences). The experiment was performed in triplicate. Data are reported as the mean values ± SE.

## RESULTS AND DISCUSSION

### Establishment of TKI-Resistant Sublines of *BCR-ABL1*-Positive MOLM-7 and CML-T1 Cells

**Figure 2.**
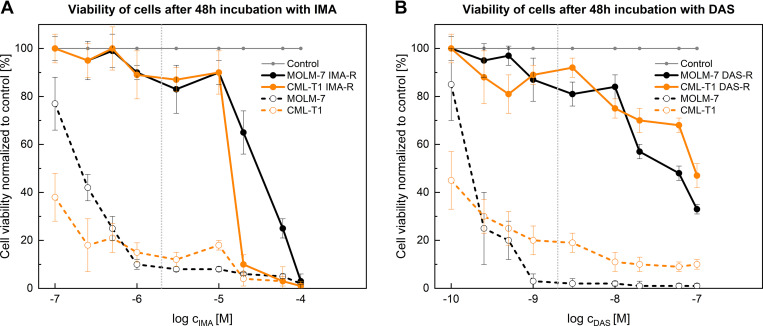
Viability of cells after 48 h of incubation with TKIs. Viability of cells after 48 h of incubation with IMA (*A*); Viability of cells after 48 h of incubation with DAS (*B*). Light gray dotted lines represent thresholds corresponding to concentrations of 2 µM IMA and 2 nM DAS, at which TKI-resistant cells were further maintained. Data represent mean values ± SE (*n* = 3). DAS, dasatinib; IMA, imatinib; TKIs, tyrosine kinase inhibitors.

Systematic exposure of parental *BCR-ABL1*-positive cell lines to increasing concentrations of IMA and DAS led to the selection of MOLM-7 and CML-T1 cells that were resistant to the tested TKIs within a specific concentration range, as depicted in Fig. 2. According to this concentration range, TKI-resistant MOLM-7 and CML-T1 cells were maintained in the presence of either 2 µM IMA or 2 nM DAS and later used for functional testing of ASP210.

### Molecular Cytogenetics of TKI-Resistant Cell Lines

Parental and TKI-resistant MOLM-7 and CML-T1 cell lines were further characterized in terms of cytogenetic changes to identify chromosomal aberrations, with special focus on the assessment of *BCR-ABL1* copy number. Multicolor FISH revealed complex chromosomal rearrangements in all cell lines (Supplemental Table S1). Representative images of mFISH are shown in Supplemental Figs. S1–S6. Polyploidy was a common finding. Both the parental and TKI-resistant sublines shared the same complex chromosomal rearrangements. FISH, but not mFISH, revealed a cryptic copy of *BCR-ABL1* on two chromosomes 22 in all CML-T1 cell lines. Four, eight, and four copies of *BCR-ABL1* were identified in diploid parental, tetraploid IMA-R, and diploid DAS-R MOLM-7 cells, respectively.

Cytogenetic analysis confirmed an increased *BCR-ABL1* oncogene copy number in all cell lines compared with standard CML (one copy); however, the copy number did not differ between the TKI-sensitive parental lines and the derived TKI-resistant sublines, suggesting that the contribution of increased oncogene copy number to the acquired resistance is unlikely.

### Sequencing of *BCR-ABL1* Kinase Domain Identifies the T315I Gatekeeper Mutation in TKI-Resistant Cells

Because point mutations in the *ABL1* kinase domain are the most common cause of TKI resistance, the mutation status of the *BCR-ABL1* kinase domain was investigated in the cell lines. The clinically relevant “gatekeeper” mutation T315I was identified in TKI-resistant CML-T1 sublines by Sanger sequencing (Supplemental Fig. S7). This result confirms a BCR-ABL1-dependent mechanism of acquired TKI resistance in the CML-T1 sublines. Conversely, because no mutations in the kinase domain or difference in *BCR-ABL1* copy number (see *Molecular Cytogenetics of TKI-Resistant Cell Lines*) were identified in the parental and derived TKI-resistant MOLM-7 cell lines, a *BCR-ABL1-*independent mechanism of TKI resistance is assumed in this case.

### Cellular Internalization of ASP210

**Figure 3.**
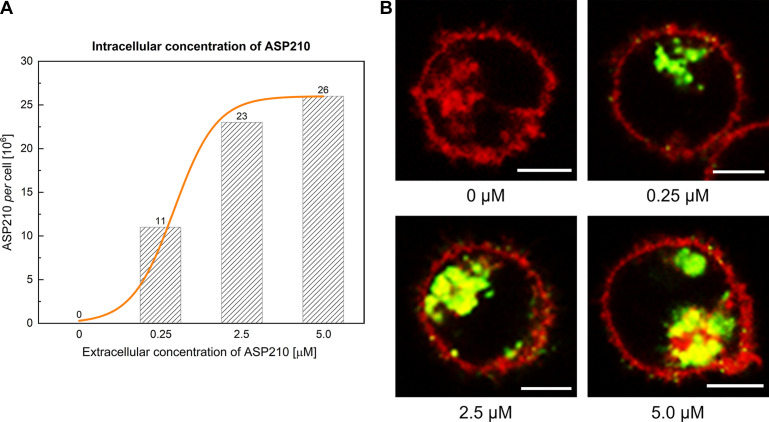
Intracellular uptake of ASP210 by IMA-R MOLM-7 cells. Amount of internalized ASP210 as function of applied concentration (*A*); Confocal microscopy images showing the distribution of fluorescently labeled ASP210 (green) applied at different extracellular concentrations (*B*). Cell membranes are shown in red. Scale bars correspond to 5 µm.

To exclude false negativity during functional studies of ASP210 in TKI-resistant sublines and relevantly evaluate its potential effects in target negative cells, it was necessary to assess the internalization of ASP210 first. Cells were exposed to increasing concentrations of ASP210 ranging from 0.25 to 5 µM to quantify the intracellular concentration of ASP210 in a 24-h time frame (Table 1). Confocal laser scanning microscopy and electrophoresis showed that the intracellular concentration of ASP210 reached a plateau at an applied concentration of 2.5 µM in all cell lines; a 2–3-fold intracellular increment was observed compared with the values at 0.25 µM, and a negligible additional increment was observed at 5 µM. Based on this finding, 0.25 and 2.5 µM ASP210 were used for dose-dependent functional validation in subsequent experiments. Representative microscopy images of IMA-R MOLM-7 cells accompanied by a graph showing the intracellular amount of ASP210 as a function of applied concentration are shown in Fig. 3.

**Table 1.** Internalization of ASP210 within 24 h

Cell Lines	Extracellular Concentration of ASP210 (µM)	Intracellular Concentration of ASP210 (molecules/cell)
MOLM-7
IMA-R	0.25	11 × 10^6^
	2.5	23 × 10^6^
	5.0	26 × 10^6^
DAS-R	0.25	7 × 10^6^
	2.5	22 × 10^6^
	5.0	24 × 10^6^
CML-T1
IMA-R	0.25	6 × 10^6^
	2.5	16 × 10^6^
	5.0	16 × 10^6^
DAS-R	0.25	5 × 10^6^
	2.5	17 × 10^6^
	5.0	18 × 10^6^
HL-60	0.25	140 × 10^6^
	2.5	236 × 10^6^
	5.0	252 × 10^6^

Notably, the amount of internalized ASP210 reached values in the order of tens to hundreds of millions of molecules per cell, an overall increase of up to 100-fold compared with values achieved with state-of-the-art oligonucleotides to date, including those tested for CML (18–21). Consistent with a previous report (22), unlike common oligonucleotides, cellular uptake was spontaneous, without the need for transfection reagents, and ASP210 was detected solely in the cytoplasm and was absent in the nucleus.

The amount of internalized ASP210 was comparable among all *BCR-ABL1*-positive cell lines [both parental (data not shown) and TKI-resistant], although it was 10-fold higher in *BCR-ABL1*-negative HL-60 cells. However, this difference in cellular internalization of ASP210 did not limit the mutual comparison of the biological effect of ASP210 between TKI-resistant and target-negative HL-60 cells. In contrast, it made it possible to monitor the potential unwanted effects of ASP210 in target-negative HL-60 cells, mimicking healthy cells, with even greater reliability.

### Reduction of *BCR-ABL1* mRNA Levels in TKI-Resistant Cells upon Treatment with ASP210

The therapeutic oligonucleotide ASP210 was designed to interfere with the e13a2 fusion *BCR-ABL1* transcript variant expressed by MOLM-7 and CML-T1 cells. TKI-resistant MOLM-7 and CML-T1 cells were treated with 0.25 or 2.5 µM ASP210 once daily for 1–10 days and incubated in parallel with untreated controls. The efficacy of ASP210 was evaluated daily as the percentage difference in the amounts of expressed *BCR-ABL1* mRNA between treated and untreated cells. Ct values assessed by RT-qPCR are summarized in Table 2 and indicate the high potency of ASP210, which decreased the levels of *BCR-ABL1* by >92% and >98% after 24 h (0.25 vs. 2.5 µM). The residual levels of *BCR-ABL1* mRNA decreased to 0% after the second dose and remained undetectable by RT-qPCR during the rest of the 10-day experiment (data not shown).

**Table 2.** Reduction of BCR-ABL1 mRNA levels in TKI-resistant cells treated with ASP210 for 24 h

	Untreated		Treated					
			0.25 µM			2.5 µM		
	Ct (*BCR-ABL1)*	Ct (*GAPDH)*	Ct (*BCR-ABL1)*	Ct (*GAPDH)*	Δ[%]	Ct (*BCR-ABL1)*	Ct (*GAPDH)*	Δ[%]
MOLM-7 IMA-R	22.26 ± 0.24	17.52 ± 0.20	30.11 ± 0.13	17.89 ± 0.08	99.44	33.83 ± 0.40	18.45 ± 0.21	99.94
MOLM-7 DAS-R	15.42 ± 0.19	21.45 ± 0.15	19.55 ± 0.15	21.77 ± 0.25	92.87	21.84 ± 0.17	21.52 ± 0.11	98.77
CML-T1 IMA-R	19.57 ± 0.21	21.85 ± 0.20	23.85 ± 0.07	22.04 ± 0.11	94.13	28.25 ± 0.31	21.70 ± 0.13	99.78
CML-T1 DAS-R	21.31 ± 0.16	19.80 ± 0.07	25.99 ± 0.38	20.03 ± 0.15	95.42	32.51 ± 0.20	19.69 ± 0.06	99.96

Ct values are expressed as the means ± SE from *n* = 5 independent experiments (each performed in duplicate). Δ stands for the % reduction of *BCR-ABL1* mRNA levels upon treatment.

The remarkably high efficiency of ASP210 in reducing *BCR-ABL1* mRNA levels compared with previous data on siRNA- or antisense oligonucleotide-mediated RNA suppression (7–13) may stem from several aspects. Our experience with multiple therapeutic oligonucleotides of this unique design (22) suggests that the combination of efficient internalization and high selectivity of ASP210 for on-target RNA over off-targets (16) plays the largest role in this. In light of the results presented here, regardless of the area of interest, oligonucleotides of this structural design could streamline a wide range of scientific and clinical applications.

### Treatment with ASP210 Significantly Decreases the Viability of TKI-Resistant *BCR-ABL1*-Positive Cell Lines

**Figure 4.**
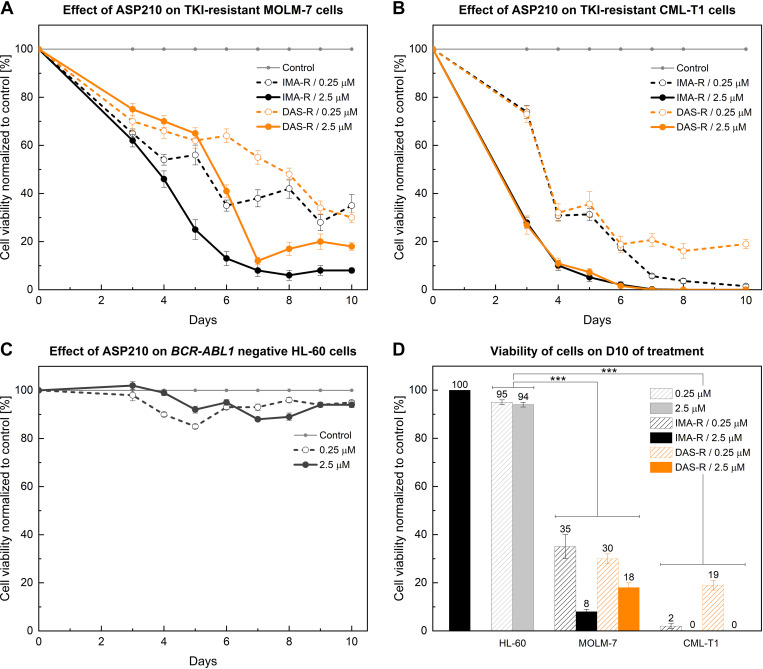
Effect of ASP210 on cell viability. *A–C*: effect of ASP210 on the viability of TKI-resistant MOLM-7 and CML-T1 cells and *BCR-ABL1*-negative HL-60 cells; (*D*) viability of cells on D10 of treatment. The Shapiro–Wilk test was used to determine normal distribution, and the statistical significance was measured by an unpaired *t* test. The same statistically significant difference *P* < 0.001 (***) was found between HL-60 cells and each TKI-resistant cell line at each of the ASP210 concentrations tested. TKI, tyrosine kinase inhibitor.

Elimination of *BCR-ABL1* mRNA as a template for leukemia driver protein was expected to affect the viability of TKI-resistant cells. As shown in Fig. 4, *A* and *B*, exposure of TKI-resistant cells to consecutive doses of ASP210 for 10 days resulted in dose-dependent progressive reduction of viability by 65%–100% after the last dose (Fig. 4*D*). In contrast, *BCR-ABL1*-negative HL-60 cells remained viable (Fig. 4*C*) despite showing 10-fold higher intracellular amounts of ASP210 (Table 1). Plots showing absolute values for each experiment are provided as Supplemental Figs. S9–S13. These results demonstrate that ASP210 is not only highly effective in suppressing the target mRNA, but it is also remarkably selective for target-positive cells. This suggests its therapeutic potential for exclusively eliminating leukemic cells while sparing healthy ones. Despite the demonstrated antileukemic effect of ASP210 on all TKI-resistant sublines tested, the decrease in cell viability differed between cell lines (MOLM-7 vs. CML-T1 as well as IMA-R vs. DAS-R). In CML-T1 cells, the decline profiles for IMA-R and DAS-R cells overlapped, and the decrease in viability was dependent only on the applied dose of ASP210. Conversely, in MOLM-7 cells, the decrease in cell viability differed between IMA-R and DAS-R cells. This difference can be attributed to the >1,700-fold higher amount (ΔΔCt = −10.77, Table 2) of target *BCR-ABL1* in DAS-R MOLM-7 cells than in IMA-R cells.

**Figure 5.**
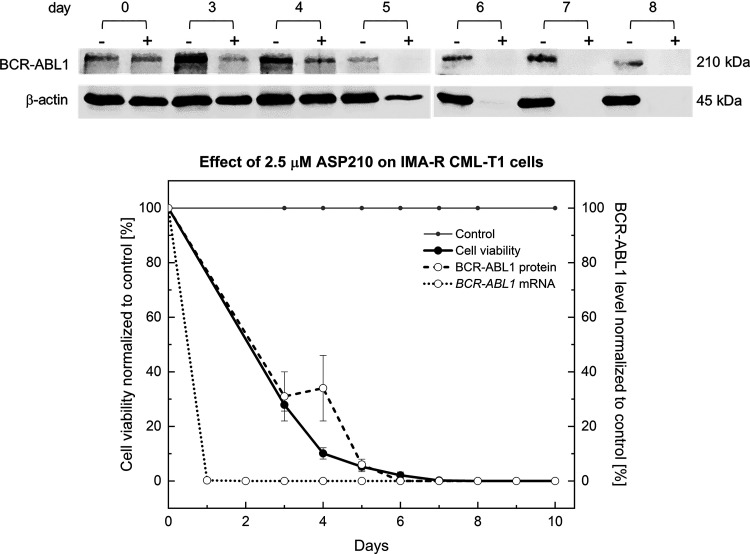
Representative profiles of reduction in *BCR-ABL1* mRNA and BCR-ABL1 protein levels as well as viability of IMA-R CML-T1 cells treated with 2.5 µM ASP210. The corresponding Western blot analysis is shown above (− stands for untreated controls, + stands for treated cells). IMA-R, imatinib-resistant; CML, chronic myelogenous leukemia.

The results also show that despite the reduction in the mRNA level to almost 0% after only 24 h (Table 2), the induced biological response in terms of the decrease in viability started with a delay in all cell lines tested (Fig. 4). This delay is related to the >40-h half-life of the BCR-ABL1 oncoprotein (8, 11, 23), the antiapoptotic activity of which maintains *BCR-ABL1*-suppressed cells viable as long as it is present. Representative Fig. 5 shows the presence of the BCR-ABL1 protein in IMA-R CML-T1 cells treated with 2.5 µM ASP210 at 30%–40% even on *days 3–4* (Supplemental Fig. S8), despite the fact that the mRNA was already suppressed on *day 1.*

Repeated daily dosing with ASP210 eventually reduced the protein to negligible levels on *day 5*. These data may also explain why researchers often failed to significantly reduce BCR-ABL1 protein levels after a single application of siRNA or antisense oligonucleotide, despite their ability to suppress the mRNA (7–13).

Overall, ASP210 eliminated the mRNA template of the driver oncoprotein BCR-ABL1 within 1–2 days and induced apoptosis of CML cells resistant to current TKI therapy, which was only delayed by the objective time required for the decay of endogenous protein.

### Perspectives and Significance

Despite excellent progress in the past two decades with the introduction of targeted molecular therapies, new strategies to address TKI resistance and reduce the leukemic burden could be a game changer in the professional management of patients with CML unresponsive to current therapies. Here, we report the results of a therapeutic oligonucleotide of original design that showed the ability to reduce *BCR-ABL1* mRNA levels with remarkable efficiency by >99% after a single application and to induce cell death in all TKI-resistant cells, including those with the clinically relevant T315I mutation, by *day 5* after redosing. The effect was selective for cancer cells, indicating a favorable safety profile for this therapeutic modality. The spontaneous cellular uptake of ASP210 and the high intracellular concentrations achieved suggest that ASP210 has the potential to be biologically effective at relatively low applied doses. The present findings suggest that ASP210 is a promising therapeutic avenue for patients with CML who fail to respond to TKI therapy.

## DATA AVAILABILITY

Data supporting the findings of this study are available from the corresponding author upon reasonable request.

## SUPPLEMENTAL DATA

10.6084/m9.figshare.25795108 Supplemental Figs. S1–S13 and Supplemental Table S1: https://doi.org/10.6084/m9.figshare.25795108.

## GRANTS

This work was supported by the Slovak Research and Development Agency under Contracts No. APVV-15–0215, APVV-19-0070, and by VEGA Grants No. 1/0069/20, 2/0160/21, and 2/0116/22. Research was further supported by the Ministry of Health, Czech Republic via project for conceptual development of research organization No. 00023736. The study was performed during the implementation of the project Building up Centre for Advanced Materials Application of the Slovak Academy of Sciences, ITMS project code 313021T081 supported by Research & Innovation Operational Programme funded by the ERDF.

## DISCLOSURES

Selecta Biotech SE holds the intellectual property rights to ASP210 (EP3362098B1). V.N. and F.R. are the inventors. All authors affiliated with Selecta Biotech SE (V.N., P.B., L.U., B.T., and F.R.) are employed by the company. A. Babelova, M.S., K.J., O.M., D.M., M.Z., A.P., A. Batorova, and L.D. have no competing interests. The funders had no role in the design of the study; in the collection, analysis, or interpretation of data; in the writing of the manuscript; or in the decision to publish the results.

Andrea Babelova is an editor of *American Journal of Physiology-Cell Physiology* and was not involved and did not have access to information regarding the peer-review process or final disposition of this article. An alternate editor oversaw the peer-review and decision-making process for this article.

## AUTHOR CONTRIBUTIONS

V.N. and F.R. conceived and designed research; P.B., B.T., L.U., A.Babelova, M.S., K.J., O.M., D.M., M.Z., and A.P. performed experiments; P.B., B.T., L.U., A.Babelova, M.S., K.J., O.M., D.M., M.Z., and A.P. analyzed data; V.N. and F.R. interpreted results of experiments; V.N. and F.R. prepared figures; V.N. and F.R. drafted manuscript; V.N. and F.R. edited and revised manuscript; V.N., A.Batorova, L.D., and F.R. approved final version of manuscript.
